# Genomic regions associated with kyphosis in swine

**DOI:** 10.1186/1471-2156-11-112

**Published:** 2010-12-21

**Authors:** Amanda K Lindholm-Perry, Gary A Rohrer, Larry A Kuehn, John W Keele, Justin W Holl, Steven D Shackelford, Tommy L Wheeler, Dan J Nonneman

**Affiliations:** 1 USDA, ARS, U.S. Meat Animal Research Center, Clay Center, NE 68933-0166, USA; 2 Smithfield Premium Genetics Group, Rose Hill, NC 28458, USA

## Abstract

**Background:**

A back curvature defect similar to kyphosis in humans has been observed in swine herds. The defect ranges from mild to severe curvature of the thoracic vertebrate in split carcasses and has an estimated heritability of 0.3. The objective of this study was to identify genomic regions that affect this trait.

**Results:**

Single nucleotide polymorphism (SNP) associations performed with 198 SNPs and microsatellite markers in a Duroc-Landrace-Yorkshire resource population (U.S. Meat Animal Research Center, USMARC resource population) of swine provided regions of association with this trait on 15 chromosomes. Positional candidate genes, especially those involved in human skeletal development pathways, were selected for SNP identification. SNPs in 16 candidate genes were genotyped in an F2 population (n = 371) and the USMARC resource herd (n = 1,257) with kyphosis scores. SNPs in *KCNN2 *on SSC2, *RYR1 *and *PLOD1 *on SSC6 and *MYST4 *on SSC14 were significantly associated with kyphosis in the resource population of swine (*P *≤ 0.05). SNPs in *CER1 *and *CDH7 *on SSC1, *PSMA5 *on SSC4, *HOXC6 *and *HOXC8 *on SSC5, *ADAMTS18 *on SSC6 and *SOX9 *on SSC12 were significantly associated with the kyphosis trait in the F2 population of swine (*P *≤ 0.05).

**Conclusions:**

These data suggest that this kyphosis trait may be affected by several loci and that these may differ by population. Carcass value could be improved by effectively removing this undesirable trait from pig populations.

## Background

Spine curvature defects have been reported in swine herds for the past three decades [1-5]. These defects have been classified as lordosis and/or kyphosis phenotypes. These reports describe this condition as a defect that can be detected visually in the live animal. We have detected a skeletal defect in both a commercial F2 and a resource herd of swine where the curvature of the spine is readily detectable in the animal carcass, but not easily identifiable externally on the live animal.

Prior literature has attributed these skeletal defects to environmental conditions rather than to genetic factors [1-5]. However more recently, Holl et al. [6] showed that the skeletal condition detected in the swine herds in this study has a genetic component with heritability estimates of 0.30 in a F2 population of animals and 0.32 in the USMARC resource population. Holl et al. [6] further reported that age, sex, number of ribs, number of lumbar vertebrae, number of nipples, carcass length and hot carcass weight were not significantly associated with the kyphosis phenotype.

Since the pig kyphosis condition is a moderately heritable trait, the purpose of this study was to identify positional candidate genes in chromosomal regions found to be associated with this kyphosis condition and to ultimately to eliminate this defect and improve carcass quality. The candidate genes evaluated in this study were: cadherin 7 (*CDH7*) and cerberus 1 homolog (*CER1*) on SSC1, cartilage oligomeric matrix protein (*COMP*), potassium intermediate/small conductance calcium-activated channel, subfamily N, member 2 (*KCNN2*) and solute carrier family 26 member 2 (*SLC26A2*) on SSC2, son of sevenless homolog 1 (*SOS1*) on SSC3, proteasome (prosome, macropain) subunit, alpha type, 5 (*PSMA5*) and Lix1 homolog (mouse)-like (*LIX1L*) on SSC4, homeobox C8 (*HOXC8*) on SSC5, ADAM metallopeptidase with thrombospondin type 1 motif, 18 (*ADAMTS18*), ryanodine receptor (*RYR1*) and lysyl hydroxylase (*PLOD1*) on SSC6, cullin 7 (*CUL7*) and runt-related transcription factor 2 (*RUNX2*) on SSC7, SRY-box 9 (*SOX9*) on SSC12 and histone acetyltransferase (monocytic leukemia) 4 (*MYST4*) on SSC14 (Table 1).

**Table 1:** Comparative human and swine map for the location of kyphosis positional candidate genes and their associated human skeletal disorders

Gene	SSC position (cM)^1^	Human position (bp)^2^	Human disorder
CDH7	1:74	18:63,418,157	
CER1	1:81	9:14,719,734	Bone mass density and fracture in Southern Chinese women [39]
COMP	2:63	19:18,893,584	Pseudoachondroplasia with vertebral anomalies and osteoarthritis [8]
PMSA5	4:115	1:109,944,478	
KCNN2	2:88	5:113,769,227	
SLC26A2	2:131	5:149,340,300	Diastrophic dysplasia with scoliosis [7]
SOS1	3:82	2:39,208,692	Noonan Syndrome [40,41]
LIX1L	4:82	1:145,477,085	
HOXC8	5:72	12:54,402,890	Cartilage defects [9]
ADAMTS18	6:8	16:77,316,026	Bone mass candidate gene [13]
RYR1	6:77	19:38,924,340	Spondylocostal dysostosis and minicore myopathy with ophthalmoplegia [10,11]
PLOD1	6:83	1:11,994,746	Kyphoscoliotic subtype of Ehlers-Danlos syndrome VIA [12]
CUL7	7:70	6:43,005,356	3 M Syndrome [14]
RUNX2	7:74	6:45,296,054	Cleidocranial Dysplasia [15]
SOX9	12:25	17:70,117,161	Campomelic dysplasia with skeletal anomalies [16]
MYST4	14:81	10:76,586,379	

^1 ^Position based on the USMARC linkage map and the RH Map [42]. SSC is the porcine chromosome.

^2 ^Position based on Feb. 2009 GRCh37/hg19 human genome sequence.

Causative mutations for human back defects have been identified in several of these genes (Table 1). *SLC26A2 *is mutated in diastrophic dysplasia with scoliosis [7]. Pseudoachondroplasia with symptoms of vertebral anomalies can be caused by mutations in *COMP *[8]. The over expression of *HOXC8 *has been shown to cause cartilage defects [9]. Mutations in *RYR1 *have been associated with the human conditions spondylocostal dysostosis (autosomal recessive) and minicore myopathy with ophthalmoplegia, both of which include curvature of the spine [10,11]. *PLOD1 *mutations are responsible for the kyphoscoliotic subtype of Ehlers-Danlos syndrome VIA (EDS VIA) [12]. *ADAMTS18 *was recently identified in a genome-wide association study as a bone mass candidate gene for human ethnic groups [13]. Mutations in *CUL7 *cause 3 M Syndrome in which one symptom is tall vertebral bodies [14]. Cleidocranial dysplasia that can include the clinical features of scoliosis or vertebral malformation is caused by mutations in the transcription factor *RUNX2/CBFA1 *[15]. Mutations in *SOX9 *have been correlated to a campomelic dysplasia with skeletal anomalies [16]. *CER1 *has recently been associated with bone mass density and fracture in Southern Chinese women, as well as in normal bone development or metabolism in the mouse [17].

## Results

### Whole genome association in USMARC resource population

A whole genome association was performed to identify chromosomal regions of interest related to the kyphosis phenotype. Markers (170 SNPs and 28 microsatellites) were located across the pig genome (Additional file 1). Whole genome association tests of these markers in the USMARC resource population of animals (n = 1,257) revealed 35 markers with association on 15 different chromosomes (Table 2). Seven of these regions, located on SSC1, 2, 5, 7, 12, and 14, contained more than one marker within a 10 cM region with significant association; the others contained one SNP or microsatellite with association with reportable significance (*P *< 0.05; Table 2). Because the purpose of the association was to identify regions associated with kyphosis, only nominal *P*-values were used to investigate these regions for candidate genes.

**Table 2:** Significant markers from the whole genome marker association in USMARC resource population with kyphosis scores

SSC^1^	Position (cM)^2^	Marker Number^3^	Marker Name^4^	df	F-ratio	*P*-value	Accession Number	dbSNP	GenBank Numbers for Microsatellite Markers
1	19	21809	13649_05	1	11.14	0.0009	BV103417	23130497	
1	20	21711	ESRA1665	2	7	0.001			Z37167, AF034972^5^
1	20	21893	ESRA1755	2	5.24	0.0054			Z37167, AF034972^5^
1	85	10237	SW780	8	2.88	0.0036			AF235365
2	60	21713	44785_574	1	4.33	0.0377	BV677845	48398076	
2	63	21788	13641_2	1	13	0.0003	BV103646	23130499	
2	63	21770	13943_1	1	5.84	0.0158	BV103450	23130298	
2	64	21725	12801_1	2	3.77	0.0234	BV103561	23129637	
2	68	21813	7241_1	2	5.72	0.0034	GF110746	262803425	
2	76	21776	41646_663	2	4.22	0.015	BV677807	48397985	
2	76	21878	M20160_638	2	3.81	0.0225	M20160^6^		
2	77	21700	23795_1	2	3.22	0.0404	BV103090	23131799	
2	131	21593	16961_1	2	11.07	0.00002	BV103264	23130997	
3	82	10112	SW142	9	3.22	0.0007			AF235215
4	33	21859	31579_1	2	5.88	0.0029	BV102560	23132873	
5	91	21597	2928_3	1	6.49	0.011	BV677897	48398268	
5	95	21596	39683_2	2	3.95	0.0196	BV677924	48398449	
6	87	21618	16951_1	2	5.91	0.0028	BV103254	23130911	
6	110	11450	APR18	3	2.75	0.0417			CU929641
7	50	21640	13438_1	2	3.35	0.0355	BV103643	23130590	
7	70	21621	14325_1	2	6.32	0.0019	BV103461	23130318	
7	70	21642	21351_2	2	2.95	0.0528	BV102746	23131826	
8	12	21592	16873_2	2	5.73	0.0034	BV103219	23130680	
8	105	21864	8975_1	2	3.01	0.0497	GF110747	262803426	
10	68	21635	17429_1	2	3.32	0.0366	BV102802	23131855	
12	0	21626	14367_1	2	4.02	0.0182	BV103479	23130349	
12	6	10965	SO143	7	2.67	0.0097			EF172262
12	26	21619	3819_2	1	11.24	0.0008	BV677898	16338405	
13	103	15490	SN287	2	4.44	0.012			X79916
14	78	21639	13687_2	2	3.22	0.0404	BV103587	23130195	
14	85	10267	SW1081	9	2.02	0.0343			AF235187
16	70	21814	7633_1	2	3.24	0.0396	GF110748	262803427	
17	18	21773	9494_161	1	3.87	0.0494	GF110745	262803424	
17	51	21722	42823_646	2	3.85	0.0216	BV677829	48398034	
18	25	21819	LEP2845	2	3.92	0.0201			AF026976^7^

^1 ^SSC is the porcine chromosome.

^2 ^Position in centimorgans based on the USMARC linkage map and the RH Map [42].

^3 ^Number of the marker in the USMARC database.

^4 ^Name of the marker in the USMARC database. Microsatellites can be found in the UniSTS database.

^5 ^SNP described in Munoz et al. [43].

^6 ^SNP described in Ciobanu et al. [44].

^7 ^SNP described in Kennes et al. [45]

Candidate genes in several regions could be identified by location, gene function and/or possible role in a human skeletal disorder. Candidate genes that were identified and selected for this study in these regions included: *CDH7 *and *CER1 *on SSC1; *COMP, KCNN2 *and *SLC26A2 *on SSC2; *PSMA5 *and *LIX1L *on SSC4; *SOS1 *on SSC3; *HOXC8 *on SSC5; *PLOD1, RYR1 *and *ADAMTS18 *on SSC6; *CUL7 *and *RUNX2 *on SSC7; *SOX9 *on SSC12; and *MYST4 *on SSC14. Table 1 describes the locations of these candidate genes in both the pig and the human and their role, if any, in human back curvature disorders.

### Positional candidate gene SNP associations in the USMARC population

SNPs were identified in candidate genes for genotyping assays by sequencing twelve animals of 8 different sire lines with all 4 of the kyphosis category scores represented. When possible, the coding regions of these genes were sequenced to identify potential mutations. A total of 195 SNPs were detected in the 16 candidate genes. The markers and their genomic positions are shown in Additional file 2.

Of the SNPs identified in the candidate genes, 70 were genotyped in the USMARC resource population. Association analysis using MTDFREML detected 10 SNPs located in *KCNN2, RYR1*, *PLOD1*, and *MYST4 *with significant association (*P *≤ 0.05) with kyphosis (Table 3). The most significant markers *(P *≤ 0.005) were in *PLOD1 *(markers 65451_236 and 65458_588).

**Table 3:** Candidate gene SNP marker associations and estimated effects in the USMARC resource population of swine

SSC^1^	Position^2^	Gene^3^	Marker^4^	Additive effect	Dominance effect	*P*	Correction^5^
2	120873345	*KCNN2*	65626_380	0.0585197	0.08578508	0.01751	NS
6	32562176	*RYR1*	67575_125	-0.1615369	-0.1089273	0.006965	NS
6	32721576	*RYR1*	68805_283	-0.0478559	-0.1008613	0.017855	NS
6	64274674	*PLOD1*	69958_99	-0.072519	-0.0770903	0.023685	NS
6	64290502	*PLOD1*	65451_67	-0.1302993	-0.0040416	0.010667	NS
6	64290571	*PLOD1*	65451_236	-0.150833	-0.2602181	0.000319	0.02
6	64291351	*PLOD1*	65458_515	0.1137805	-0.0142219	0.020264	NS
6	64291424	*PLOD1*	65458_588	0.0831903	-0.1026598	0.002483	NS
6	64291439	*PLOD1*	65458_603	-0.1545773	0.03408042	0.014415	NS
14	80811196	*MYST4*	12267_169	0.1447504	-0.0619209	0.040538	NS
							

^1 ^SSC is the swine chromosome.

^2 ^Position based on the pig genome sequence assembly Nov. 2009 (SGSC Sscrofa9.2/susScr2).

^3^Candidate gene or gene located nearest the amplicon.

^4 ^Name of the marker in the USMARC database.

^5 ^The correction for multiple tests was performed by multiplying *P *from the association analysis with the number of markers (n = 70).

Six synonymous SNPs were discovered in the coding regions of *PLOD1*; these were located in exons 1, 5, 7, and 11. Another SNP was also identified in the 3' untranslated region of the gene (Additional file 2). Of the seven SNPs in the *PLOD1 *coding region, six were genotyped in the USMARC resource population of animals and two were analyzed (Table 3). Four were excluded from the analysis due to high numbers of genotyping errors. One of the coding SNP located in exon 11 of *PLOD1*, marker 65451_67, was significant for the kyphosis defect (*P *= 0.01; Table 3). Five additional markers located in introns 1, 11 and 14 of *PLOD1 *were significant for association with kyphosis in this population of animals. Two of these markers were significant at *P *< 0.005; they were markers 65451_236 in intron 11 (*P *= 0.00032) and marker 65458_588 in intron 14 (*P *= 0.0025; Table 3). The estimated additive effects of markers 65451_67, 65451_236, and 65458_588 were 0.13, 0.151, and 0.083, respectively.

### Positional candidate gene SNP associations in the F2 population

Forty-seven SNPs in candidate genes were recombined into assay groups and genotyped across the F2 population of animals. These assays included SNPs that were significant in the USMARC population of animals. Nine were significantly associated (*P *≤ 0.05) with the kyphosis defect (Table 4). These SNPs were located in *CER1*, *CDH7, ADAMTS18, PSMA5*, *HOXC6*, *HOXC8 *and *SOX9*. The most significant markers *(P *≤ 0.005) were in *CDH7 *(marker 65672_206), *HOXC6 *and *HOXC8 *(markers 44017_483 and 44360_103, respectively) and *SOX9 *(marker 65525_208).

**Table 4:** Candidate gene SNP marker associations and estimated effects in the F2 population of swine

SSC^1^	Position^2^	Gene^3^	Marker^4^	Negative allele	Effect	Positive allele	Effect	*P*	Correction^5^
1	37034220	*CDH7*	65672_206	G	-0.075	A	0.0753	0.0051	NS
1	153877484	*CER1*	65678_443	T	-0.131	C	0.131	0.0341	NS
4	115208461	*PSMA5*	65650_316	A	-0.084	G	0.0842	0.0059	NS
5	16484535	*HOXC6*	44017_483	G	-0.085	A	0.0847	0.001	0.05
5	16484123^6^	*HOXC8*	44360_103	C	-0.082	A	0.082	0.0017	0.08
6	7627094	*ADAMTS18*	65640_162	C	-0.093	G	0.0931	0.0491	NS
12	6871500	*SOX9*	65517_61	C	-0.088	T	0.0875	0.012	NS
12	6973909	*SOX9*	65525_208	G	-0.088	C	0.0881	0.0045	NS
12	6973990	*SOX9*	65525_389	T	-0.068	C	0.0682	0.0295	NS

^1 ^SSC is the swine chromosome.

^2 ^Position based on the pig genome sequence assembly Nov. 2009 (SGSC Sscrofa9.2/susScr2).

^3 ^Candidate gene or gene located nearest the amplicon.

^4 ^Name of the marker in the USMARC database.

^5 ^The correction for multiple tests was performed by multiplying the *P *from the association analysis with the number of markers (n = 47).

^6 ^Position based on BAC End sequence.

Twelve SNPs in the *SOX9 *gene that were genotyped in the USMARC resource population of animals were not significantly associated with kyphosis. Three *SOX9 *SNPs (markers 65517_61, 65525_208 and 65525_389) were genotyped in the F2 population of animals and all were significant for the back defect (*P *< 0.03; Table 4). Marker 65517_61 is located in the 5'UTR of the gene and the other two markers are located in intron 2. The effects of these SNPs on the kyphosis phenotype ranged from ± 0.0682 to ± 0.0881 (Table 4).

The SNP associated with kyphosis in *CER1 *(marker 65678_443) is a polymorphism responsible for a nonsynonymous amino acid alteration in exon 1 (Table 4). In total, three polymorphisms were identified in the coding regions of *CER1*. Two SNPs were identified in exon 1 of *CER1*; a nonsynonymous alteration responsible for an Arg to His change at amino acid 24 in the human sequence (marker 65678_419) and a nucleotide change causing a Phe to Ser amino acid change at position 32 (marker 65678_443). In addition, a silent base change was identified in the codon for Pro at amino acid 120 (Additional file 2). Marker 65678_443 was significant for kyphosis in the F2 population of swine (*P *= 0.034) and displayed an estimated effect of ± 0.131 (Table 4). This SNP was not associated with the back defect in the resource population.

A joint analysis of the markers genotyped in the two swine populations resulted in four markers that were significant. Markers 65451_236, 65458_515, 65517_61 and 65525_208 in *PLOD1 *and *SOX9 *were associated with nominal *P *of 0.02, 0.001, 0.02, and 0.05, respectively (data not shown).

The number of significant SNPs (*P *< 0.05) detected in these populations (14% in the resource population and 19% in the F2 population) exceeded the chance expectation of 5% (Tables 3 and 4). The FDR based on *P *= 0.05 for the resource population of swine is 0.35 and 0.26 for the F2 population of swine, suggesting that 35% of the associations detected are expected due to chance in the resource population and 26% were expected due to chance in the F2 population. The FDR based on *P *= 0.01 is 0.23 for the resource population and 0.094 for the F2 population.

A correction for multiple testing was applied to the SNPs significant for kyphosis. Markers were corrected using a Bonferroni calculation by multiplying the *P*-values by the number of SNPs genotyped in each population. One marker in *PLOD1 *remained significant (*P *= 0.02) in the resource population and one marker in *HOXC8 *remained significant (*P *= 0.05) in the F2 population (Tables 3 and 4).

## Discussion

The kyphosis detected in the two swine populations evaluated in this paper and in Holl et al. [6], does not seem to be the same as the kyphosis conditions that were previously reported for swine in the literature [1-5]. It is difficult to detect the kyphosis defect in the live animal in the populations presented here and the curvature does not appear to be the result of hemivertibrae. The vertebrae in our swine herds appear to be of typical rectangular shape and curvature of the spine does not appear to be the result of ossification defects (data not shown).

The kyphosis condition presented in this paper also appears to differ from the human scoliosis condition, Scheuermann's disease. Vertebrae in subjects with Scheuermann's are curved due to ossification deficiencies that result in a wedge shape, which does not seem to be present in these swine herds. In addition, the mode of inheritance of Scheuermann's suggests an autosomal dominant heritability with complete penetrance in males and incomplete penetrance in females [18], while sex is not significantly associated with the condition presented here [6].

In humans, kyphosis is often one of a multitude of symptoms of a disorder. For example, kyphosis can be a symptom in Rett Syndrome, but the disease is characterized as a neurodevelopmental disorder and is detected almost exclusively in girls. Other symptoms include regression of higher functions, loss of speech, stereotypical movement of the hands, microcephaly, and mental retardation [19,20]. Another example is Larsen Syndrome [21], caused by mutations in filamin B, where individuals can display kyphosis; however, other symptoms of the disease include dislocation of the knees, flat facial features, and cylindrical-shaped fingers. These herds of swine do not display neurological, vocalization loss, facial and/or developmental defects; the only noticeable symptom has been the spinal curvature.

While there are differences between known human back curvature defects, there are also some similarities. This condition displays a genetic mode of inheritance and a similar physical S curvature of the spine in all animals evaluated. In addition, the kyphosis condition described in the Duroc-Landrace-Yorkshire resource population was not detectable in neonatal animals (data not shown). Neonatal animals (n = 30) of parents known to pass along this kyphosis defect were harvested at 110 days of gestation and radiographed to determine whether the kyphosis condition could be identified early in these animals. The kyphosis was not detected in any of the neonatal animals examined. This condition may be similar to back curvature defects in humans that do not present until childhood or adolescence.

Several chromosomal regions were identified with a whole genome association analyses (Table 2). These regions were investigated further for genes that may be involved in the back defect via bone development pathways or known involvement with human spine defects. The development of vertebrae occurs through the reorganization of the somites that are formed on both sides of the midline neural tube during embryonic development [22]. The somites are reorganized into sclerotomes that ultimately give rise to the vertebrae. There are three phases of somite development during embryogenesis which includes a membraneous phase, a chondrification phase and an ossification phase [22]. Somite patterning for vertebrae specification is determined by the expression of the HOX gene family along the craniocaudal axis [22].

Two SNPs in the *HOXC *cluster of genes were identified as significant in the F2 population of animals (Table 4). The HOX genes are transcription factor clusters that control the positional architecture of an organism's body layout. Each HOX cluster (i.e., *HOXA*, *HOXB*, *HOXC *and *HOXD*) is located on a different chromosome and the genes in each cluster are thought to be the result of tandem duplications [23]. The number of the HOX gene describes the location of its anteroposterior axis expression with smaller gene numbers expressed closer to the head of the animal and larger numbers are expressed further down the spinal column. The *HOXC8 *and *HOXD8 *genes are involved in patterning the lower thoracic and lumbar vertebrae [24]. One of the significant SNP in this study is located in *HOXC8*, which is normally expressed during embryogenesis to reorganize the somites [22,24] and also in chondrocytes [9]. When overexpressed in chondrocytes, *HOXC8 *causes cartilage defects characterized by an abundance of immature, proliferating chondrocytes. The normal function of *HOXC8 *is to act on skeletal development by influencing the maturation of chondrocytes along the differentiation pathway [9].

SNPs in *SOX9 *were significant in the F2 population. *SOX9 *is a master transcriptional regulator of chondrogenesis [25]. Mutations have been identified in *SOX9 *that cause haploinsufficiency and result in the human clinical condition campomelic dysplasia (CMD1). CMD1 is a skeletal condition often associated with XY sex reversal. Clinical phenotypes of this disorder include bowing and angulation of the long bones, short stature and anomalies of vertebrae and the spine [16]. *SOX9 *has also been shown to bind to regulatory regions of *RUNX2 *repressing its activity *in vitro *[26]. In CMD1 cartilage with heterozygous *SOX9 *mutations, *RUNX2 *expression was upregulated due to loss of repression [26]. We also evaluated *RUNX2 *in this study; however, we did not detect a significant association between the swine kyphosis condition in the two populations tested. Additional SNPs in the region of *RUNX2 *will need to be examined to rule out involvement of *RUNX2 *in this phenotype.

In addition to genes that may be involved with vertebral development pathways, genes that play a role in bone structure or bone growth were also investigated. Bone consists of osteoblasts that form bone, osteoclasts that remove bone tissue and osteocytes that are mature osteoblasts no longer capable of forming bone [27]. In addition, bone includes other fibers and compounds that help strengthen the bones; these consist mainly of collagen and minerals. The function of collagen is to give the bone tensile strength to allow the bone to withstand stretching forces [28]. The processes of producing, growing and maintaining bone and vertebrae are controlled by multiple genes and pathways.

*PLOD1 *SNPs were significantly associated with the back defect in the resource population of swine examined in this study. *PLOD1 *is an enzyme that forms hydroxylysine on collagen. Over 20 different mutations in the *PLOD1 *gene are responsible for Ehlers-Danlos Syndrome VIA (EDS VIA) [12], which is the kyphoscoliosis type of this connective tissue disorder. These mutations include a large duplication, point mutations that alter the amino acid sequence, mutations that result in a truncated protein, and splice site mutations that cause exons to be skipped [12]. Lysyl hydroxylase deficiency results in the common symptoms of muscle hypotonia, newborn joint laxity and kyphoscoliosis.

We did not identify any potentially detrimental mutations in the coding regions of *PLOD1 *or *SOX9*; however, we did discover several SNPs located close to intron junctions in *PLOD1*. It is also possible that in swine, the mutations in these genes may be located in promoter regions responsible for gene expression. While the majority of the human mutations in *PLOD1 *seem to involve a large duplication or tend to be located in the coding regions of the gene, the phenotypic expression of the disorder is also much more severe than the phenotype we have seen in these swine populations. Alternatively, it is also possible that we have not identified the correct positional candidate gene for kyphosis in these regions and that the SNPs in this study are simply in LD with the causative genes.

## Conclusions

A total of 19 markers in 9 positional candidate genes were significantly associated with the skeletal defect; however, none of the same markers were detected in both the F2 and the USMARC resource populations of animals. These data suggest that the skeletal defect found in these swine herds is under polygenic control and that these populations are not segregating the same variation in these regions. Thus, there are likely to be additional SNPs in these and other genes that likely play a role in the kyphosis phenotype in swine. These data indicate that to eliminate this condition using genetic selection will involve screening SNPs in multiple genes and developing more predictive markers. The significant markers account for about 1% of the phenotypic variation in kyphosis present in these two commercial-like populations of swine. However, these markers must be evaluated in other commercial populations of swine with kyphosis before being used for genetic selection.

## Methods

### Animal care

Animals were fed ad libitum a corn-soybean meal grower ration of 18% protein from 8 to 13 wk, 16% from 13 to 16 wk of age and 15% protein after 16 wk of age [29]. Diets were formulated to meet or exceed National Research Council recommendations [30]. All animal procedures were reviewed and approved by the U.S. Meat Animal Research Center Animal Care and Use Committee and procedures for handling pigs complied with those specified in the Guide for the Care and Use of Agricultural Animals in Agricultural Research and Teaching [31].

### F2 research and USMARC resource populations and phenotyping

The F2 population of swine was produced from 4 F1 boars and 50 F1 females. The F1 parents were reciprocal crosses between purebred Duroc and Landrace pigs and were raised indoors at a commercial production site under commercial production conditions. At approximately 114 kg, pigs were harvested at a commercial facility where they were processed [32]. These animals were phenotyped at USMARC 24 hours after harvest.

USMARC resource animals are from a multigenerational population developed by mating Yorkshire-Landrace composite females to either Duroc or Landrace boars selected from the industry. Twelve boars of each breed were mated to Yorkshire-Landrace females (n = 220) and further developed by reciprocally mating female descendants to either a Duroc or Landracesired boar. Subsequent *inter se *matings were random except that matings of the same sire line were avoided [33]. All animals were housed in indoor facilities on concrete floors and plastic slats. These animals were harvested at the USMARC abattoir and phenotypes were collected as described in Holl et al. [6]. Sows (n = 398) were harvested after first or second parity; the other animals were market age hogs. Briefly, subjective back scores were given for each spine evaluated in the split carcass. One score was given per animal by one trained individual per population of swine. Four categories were used: normal (0), mild (1), moderate (2), and severe (3). Mild was defined as a slight convex curvature of vertebrae. Moderate was defined as a convex curvature in the vertebrae and spinal cord. Severe was defined as an extreme convex curvature of the vertebrae and spinal cord [6]. Of the animals phenotyped in F2 and USMARC populations, 75.6 and 68.9% were normal (0), 11.1 and 23.3% were mild (1), 11.1 and 6.2% were moderate (2), and 2.2 and 1.5% were severe (3), respectively [6].

### SNP discovery

Primer pairs for amplification of candidate genes were designed from available porcine EST, genomic, BAC-end, and BAC clone sequences deposited in GenBank using Primer 3 [34] (code available at http://frodo.wi.mit.edu/primer3/). Primer sets were obtained from IDT (Integrated DNA Technologies, Coralville, IA). PCR was performed in a Dyad PTC-0220 DNA engine (MJ Research Inc., Watertown, MA) using 0.5 U HotStar *Taq *polymerase (Qiagen, Valencia, CA); 1 × supplied buffer; 1.5 mM MgCl_2_; 200 μM dNTPs; 0.8 μM each primer; and 50-100 ng genomic DNA of 12 animals representing 8 different sire lines with all of the kyphosis scores represented, in 25 μl reactions. Five microliters of the PCR reaction was electrophoresed in 1.5% agarose gels to determine quality of amplification, and the remainder was prepared for sequencing after treatment with 0.1 U exonuclease I (USB, Cleveland, OH). Sequencing reactions were precipitated with 58% isopropanol and sequenced with an ABI 3730 capillary sequencer (Applied Biosystems, Foster City, CA). Bases were called with Phred and assembled into contigs with Phrap. Polymorphisms were identified using Polyphred and assessed using Consed http://www.phrap.org.

### Genotyping analysis

Multiplex assays for use in the Sequenom MASSARRAY^® ^system were designed using MASSARRAY^® ^Assay Design software v3.1 (Sequenom, San Diego, CA). Each amplification primer had a 10-base tag added to ensure that the amplification primer masses were outside the range of the allele masses. Amplicon lengths were approximately 120 bp. Reaction conditions were performed as suggested by Sequenom iPLEX chemistry.

### Whole genome association analyses

The resource population of animals (n = 1,257) was genotyped for 198 markers (28 microsatellites and 170 SNPs) dispersed throughout the genome to find chromosomal regions with significant association with the kyphosis phenotype. A single marker association was performed using the programs GenoProb and MTDFREML [35,36]. The model included fixed effects of sex and harvest date, random additive polygenic effects, and SNP were fitted as a covariate (e.g., number of copies of an allele). Regressors for additive and dominance effects of each SNP were calculated using genotypic probabilities generated by allelic peeling algorithms in GenoProb [35] as described by Kuehn et al. [37]. Variance components were estimated using MTDFREML [36]. Each SNP was fitted separately due to potential multi-colinearity between regressions of closely linked markers. Nominal significance values were computed.

### SNP association analysis (USMARC resource population)

The genotypic data and pedigree from the resource population of animals (n = 1,623) were analyzed using GenoProb and MTDFREML with the same animal model described above for the whole genome association analysis.

### SNP association analysis (F2 population)

The genotypic data from the F2 population (n = 371) of animals were analyzed for association with the skeletal defect using Model 1 of the QTL Association option of Mendel 8.0.1 [38]. The program utilizes full pedigree and the model included the covariates (or predictors) of age and sex, and the contemporary groups (or household effects) of year and season. Covariance classes analyzed were additive, dominant and environmental.

### False Discovery Rate (FDR)

The FDR was calculated for each population of animals with the following equation:

FDR=(#SNPs Tested×P-value)/#Significant SNPs

## Authors' contributions

ALP carried out the molecular genetic studies, participated in the design of the study and drafted the manuscript.

GAR collected the phenotypes on the USMARC resource population, conceived the study and participated in the project design and performed statistical analyses.

JWK, LAK and JWH performed statistical analysis of genotypic data.

SDS and TLW identified the kyphosis condition in the commercial population of animals and collected and provided phenotypic data.

DJN conceived the study, participated in design and coordination and helped draft the manuscript.

All authors read and approved the manuscript.

## Supplementary Material

Additional file 1 **Genome-wide association of 198 microsatellite and SNP markers for kyphosis in swine**. Primer sequences and association analysis for 198 SNPs and microsatellites located across the swine genome [46]. Click here for file

Additional file 2 **SNPs identified in candidate genes for kyphosis in swine**. Primer sequences, location and dbSNP numbers for SNPs identified in candidate genes. Minor allele frequencies are provided for SNPs that were genotyped. Click here for file
